# The Inhibition of Microcystin Adsorption by Microplastics in the Presence of Algal Organic Matters

**DOI:** 10.3390/toxics10060339

**Published:** 2022-06-20

**Authors:** Bingran Tang, Ying Tang, Xin Zhou, Mengzi Liu, Hong Li, Jun Qi

**Affiliations:** 1 Key Laboratory of Eco-Environment of Three Gorges Region, Ministry of Education, Chongqing University, Chongqing 400044, China; tangbingrancqu@163.com (B.T.); zhouxincqu@163.com (X.Z.); liumengzicqu@sina.com (M.L.); 2 Chongqing Key Laboratory of Soil Multi-Scale Interfacial Process, Department of Soil Science, College of Resources and Environment, Southwest University, Chongqing 400715, China; yingtang@swu.edu.cn; 3 Department of Hepatobiliary Pancreatic Tumor Center, Chongqing University Cancer Hospital, Chongqing 400045, China

**Keywords:** microplastics, microcystin, intracellular organic matter, adsorption isotherm, kinetics of adsorption

## Abstract

Microplastics (MPs) could act as vectors of synthetic chemicals; however, their influence on the adsorption of chemicals of natural origin (for example, MC-LR and intracellular organic matter (IOM), which could be concomitantly released by toxic Microcystis in water) is less understood. Here, we explored the adsorption of MC-LR by polyethylene (PE), polystyrene (PS), and polymethyl methacrylate (PMMA). The results showed that the MPs could adsorb both MC-LR and IOM, with the adsorption capability uniformly following the order of PS, PE, and PMMA. However, in the presence of IOM, the adsorption of MC-LR by PE, PS, and PMMA was reduced by 22.3%, 22.7% and 5.4%, respectively. This is because the benzene structure and the specific surface area of PS facilitate the adsorption of MC-LR and IOM, while the formation of Π-Π bonds favor its interaction with IOM. Consequently, the competition for binding sites between MC-LR and IOM hindered MC-LR adsorption. The C=O in PMMA benefits its conjunction with hydroxyl and carboxyl in the IOM through hydrogen bonding; thus, the adsorption of MC-LR is also inhibited. These findings highlight that the adsorption of chemicals of natural origin by MPs is likely overestimated in the presence of metabolites from the same biota.

## 1. Introduction

Microplastics (MPs), particles of sizes less than 5 mm [1], are widely found in the environment. Recently, it was found that the amount of MPs in Lake Chaohu reached 6250 particles/m^3^ and could reach approximately 100,000 particles/m^3^ in the water [2]. It is also found that nearly 80% of the MPs are polyethylene (PE), polypropylene (PP), polyvinyl chloride (PVC), polyurethane (PUR), PE terephthalate (PET), and polystyrene (PS) [3], which are primarily associated with plastic production. Considering that MPs are pervasive in the environment, their potential impact on chemicals and biota (including human beings) has caught the public’s attention. MPs can also alter the physical properties of ultraviolet radiation, mechanical damage, and biological degradation [4].

Apart from the intrinsic properties of the MPs, the most common concern from the public is the interaction between MPs and other pollutants. Research has found that persistent organic pollutants, pharmaceuticals, pesticides, and personal care product ingredients can be adsorbed by MPs [5,6], and that the MP types, particle size, and surface properties, as well as the crystallinity of the polymer, can influence the pollutants’ adsorption [6,7]. For example, it was previously found that the surface carboxyl functional groups and H-bonding jointly regulated the adsorption of malachite green on nylon MPs [8], while the hydrophobic effect, electrostatic repulsion, and CH-π interaction forces are responsible for the adsorption of anthracene by PVC [9]. Environmental factors such as salinity, pH, and humic acid concentrations can affect the adsorption behavior of pollutants on MPs [10]. It was found that the fouling of MPs by natural organic matter, which was ubiquitous in natural waters, led to a significant reduction in their adsorption capacity [11]. Nevertheless, there are concerns that the interaction of chemicals/pollutants and MPs will result in the transport of these adsorbed pollutants and minify their potential risk towards biota [12].

It was worthy of note that studies concerning the mitigation of pollutant transport predominantly focused on the synthetic chemicals of anthropogenic origin, such as heavy metals, pharmaceuticals, and pesticides. In natural water, some chemicals can be produced and released into the water from biota in the aquatic environment. For example, the input of excessive nutrients into freshwater altered the total biomass of algal communities and may cause the occurrence of harmful algal blooms. Microcystins (MCs), which can cause liver damage and promote tumor production [13,14], can be produced by toxin-producing Microcystis strains [15] and released into the surrounding water. Generally, MCs are predominant in toxin-producing algal cells, but can be released into the water when the algal cells are lysed under stress; hence, the MC concentration in the water could reach several micrograms per liter. Previously, it was documented that MCs can be adsorbed by PS microplastics [16]. This was also confirmed in a recent study [17]. This evidence emphasizes the need to investigate the interaction between MPs and chemicals of natural origin. However, these chemicals—take MCs for example—are released from the biota cells and are associated with the secretion and excretion of algal organic matter (AOM), which is released into the water either as metabolic byproducts or due to cell lysis. AOM, including intracellular organic matter (IOM) and extracellular organic matter mainly composed of proteins and polysaccharides [18,19], is likely to influence the physicochemical properties and aggregation potential of particles [20]. The presence of AOM may prompt the adsorption on MPs as providing new active sites because of the interaction between the particle and the AOM [21], or hinder the adsorption of pollutants due to the higher affinity of the AOM toward the MPs’ surface; thus, it stands to reason that the presence of AOM is likely to regulate the adsorption of MCs by MPs and, consequently, manipulate its environmental transport. However, such information is still lacking to our best knowledge.

Thus, the current study was set out to (1) determine the adsorption capability and process of MC-LR (the most frequently detected MCs variants [22]) by PS, PE, and polymethyl methacrylate (PMMA) (all of them are widely found in freshwater systems) [23]; (2) elucidate the potential competition for binding sites between the IOM and MC-LR in contact with PS, PE, and PMMA; and (3) reveal the essence of the interaction between MPs, MC-LR, and IOM combined with the assistance of Fourier-transform infrared (FTIR) spectroscopy and X-ray photoelectron spectroscopy (XPS) analysis. The purpose of the current study was to evaluate the adsorption sorption capability of the three kinds of MPs towards the pollutants of natural origin and elucidate the potential mechanism involved. The outcomes of this study may provide a theoretical basis for the estimation of the potential of MPs to act as a vector for organic pollutants of natural origin.

## 2. Materials and Methods

### 2.1. MPs Particles, IOM Samples, and Chemicals

PE, PS, and PMMA, with a uniform size of 50 µm, were purchased from Zhongkeleiming Daojin Technology Co., Ltd. (Beijing, China). The particle size, zeta potential, crystalline compositions, morphological traits, and contact angles of the studied MPs are characterized and documented in the supporting information (Figures S1–S3). The MP solution was ultrasonicated (25 °C, 25 min, 150 W) to ensure homogeneity before use. The IOM was obtained from the non-toxic *Microcystis aeruginosa* (FACHB-469) following the methods described in the literature [24]. The MC-LR standards were purchased from Express Technology Co., Ltd. (Beijing, China). Other reagents used in the experiments were analytical reagents.

### 2.2. Batch Adsorption Experiments

In the kinetics experiments, 2 mg of the PE, PS, and PMMA were individually added into the Erlenmeyer flask containing 100 mL Milli-Q water (with an MP concentration of 20 mg/L). Then, three independent adsorption tests were performed. In the first experiment, the equivalent amount of MC-LR standard was added into each flask (containing water and MPs) to reach the toxin concentration of 400 µg/L. The solution was shaken for 48 h at 150 rpm, and the water was collected after 0 min, 30 min, 1 h, 2 h, 6 h, 12 h, 24 h, and 48 h by a 0.45 μm filter (cellulose acetate filter) to remove the MPs, and the aqueous MC-LR was quantified using an HPLC-MS system (Agilent1100, Agilent, Wilmington, DE, USA) [25]. In the second experiment, the adsorption of IOM by PE, PS, and PMMA was evaluated, with the experimental system being the same as experiment 1, but with the addition of IOM (to reach the initial concentration of 5 mg/L) instead of MC-LR. The IOM concentration (as revealed by the concentrations of DOC) in the supernatant was determined using a TOC analyzer (Shimadzu, TOC-L) at the same intervals as experiment 1. In order to evaluate the influence of IOM on the adsorption of MC-LR by MPs, the third experiment was conducted in the presence of 5 mg/L IOM and MC-LR (with the same concentration of MC-LR as the experiment 1). The Milli-Q water that contained the same concentration of MC-LR and IOM, but without the addition of MPs, was used as the control. NaN_3_ was added to eliminate the influence of microorganisms [19]. The initial pH of the solutions was adjusted using 0.1 M HNO_3_ or 0.1 M KOH solution. All of the samples were shaken in a dark and constant temperature shaker (25 ± 1 °C, 150 rpm). All samples were performed in triplicate.

The adsorption isotherm experiments were undertaken in triplicate. For the first sorption isotherms, we selected different concentrations of MC-LR (50, 100, 150, 200, 300, 400, and 500 μg/L). The same mass of PE, PS, and PMMA (20 mg/L) was transferred to each flask, respectively. The mixtures were shaken at 150 rpm for 24 h (the equilibration time was determined from the sorption kinetics experiments). Then, the aqueous MC-LR concentration was measured. The second sorption isotherm experiments were performed to assess the adsorption capability of PE, PS, and PMMA towards IOM. Specifically, 1, 2, 4, 6, 8, 10, and 12 mg/L of IOM was prepared in the flask containing the same mass of the studied MPs, respectively, and the IOM concentration in the water was detected after 24 h of incubation, shaken at 150 rpm. To identify the influence of IOM on the adsorption of MC-LR by PE, PS, and PMMA, the third experiment was conducted. The experimental condition was consistent with the first adsorption isotherm experiment, but with the addition of IOM (at a concentration of 5 mg/L and could be totally adsorbed by the MPs according to the adsorption capability of IOM by the studied MPs that were quantified from the second sorption isotherm experiment). The MC-LR concentration in the water was determined after 24 h with the same methods described in the kinetics experiments. All of the isotherm experimental conditions were consistent with the kinetics experiments. After adsorption, the PE, PS, and PMMA were collected and freeze-dried, and then underwent FTIR and XPS determination for further mechanism analysis.

### 2.3. Adsorption Model

The Pseudo-first-order (Equation (1)) and Pseudo-second-order (Equation (2)) kinetic models were used to analyze the experimental data.

(1) $$q_{t}=q_{e}\left(1-e^{-k_{1}t}\right)$$

(2) $$\frac{t}{q_{t}}=\frac{1}{k_{2}q_{e}^{2}}+\frac{1}{q_{e}}t\;\;\;\;$$

where *q_t_* is the adsorption capacity (mg/g) at time *t* (min), *q_e_* is the adsorption capacity (mg/g) at equilibrium time, *k*_1_ is the first-order rate constant (mg/g⸱min) and *k*_2_ is the second-order rate constant (mg/g⸱min).

Freundlich and Langmuir adsorption models were applied to fit the adsorption isotherms of the MC-LR, IOM, and MC-LR adsorption in the presence of IOM.

(3) $$q_{e}=\frac{q_{m}k_{a}C_{e}}{1+k_{a}C_{e}}$$

(4) $$q_{e}=\frac{q_{m}k_{f}C_{e}}{1+k_{f}C_{e}}$$

where *q_e_* and *q_m_* are the equilibrium and maximum adsorption amount, respectively. *C_e_* is the concentration pollutants at the equilibrium. *k_a_* is the Langmuir adsorption constant. *k_f_* is the Freundlich adsorption coefficient, and n is the Freundlich isotherm exponent.

### 2.4. Instrumental Analyses

The characterization of the studied MPs was performed to illustrate their surface property. Specifically, the zeta potential of the MPs in the experimental medium under pH 2.5–9 was measured using a Malvern Zetasizer instrument (Nano ZS, Worcestershire, UK). Scanning electron microscopy (SEM, Nova Nano SEM 450, FEI Co., Hillsboro, OR, USA) was used observe the surface morphology. X-ray diffraction (XRD, D8 Advance, Bruker, Germany) was adopted to detect the change in crystallinity of the MPs. The surface wettability of the MPs was detected using the contact angle meter (JC2000D, Powereach Co., Shanghai, China). X-ray photoelectron spectroscopy (XPS) spectra of the MP samples before and after the adsorption experiments were collected with a PHI5000 XPS (ULVAC-PHI, Osaka, Japan). Fourier-transform infrared (FTIR) spectroscopy (Nicolet 5700, Thermo Electron Corp., Madison, WI, USA) was chosen to characterize the change in the surface functional groups of the studied MPs. Specifically, the measurement mode and parameters were as follows: scanning range, 400–4000 cm^−1^; resolution, 4 cm^−1^; and number of scans, 64. The resulting spectra of the MPs were calibrated with the OMNIC software package (Thermo, Nicolet 380, Madison, WI, USA). The baseline was obtained by scanning spectral-grade potassium bromide pellets. The IOM composition was analyzed by three-dimensional excitation emission matrix (3D-EEM) fluorescence spectroscopy (F7000, Hitachi, Tokyo, Japan). The specific surface area was determined by N_2_-BET analysis (Micromeritics).

### 2.5. Statistical Analysis

The adsorption kinetics and isotherms were fitted using Origin 2015 software, and an analysis of variance (ANOVA) was applied. Statistical differences were considered significant at *p* < 0.05.

## 3. Results and Discussion

### 3.1. MPs Characterization

The average particle size (d_(0.5)_) of the PE, PS, and PMMA, calculated from Figure S1a, was 43.06, 49.96, and 55.47 μm, respectively. The negative values of the three studied MPs (<0 mV at pH in the range between 4.5 and 9, Figure S1b) manifested that PE, PS, and PMMA existed stably in the suspensions because of electrostatic repulsion between the particles. The degree of crystallinity of the three studied MPs followed the order of: PE (35%), PS (3.7%), PMMA (6.1%) (Figure S2). Figure S3 illustrates the SEM images of the studied MPs. It was observed that PE revealed irregular granular morphology and had relatively more dents, while PS and PMMA presented a smooth surface. It was previously found that with the increase in crystallinity, the plastics would be more fragile [26]. The maximum crystallinity of PE may lead to the fragmentation of the particles; thus, the particle surface may seem rough (Figure S3). It can also be observed from Figure S3 that the contact angles of PE and PS were 108.8° and 106.3°, respectively, indicating that the surfaces of PE and PS are hydrophobic. In contrast, the contact angles of PMMA were identified to be 85.1°, suggesting PMMA is hydrophilic. Table 1 presents the characterization of the studied MPs and clearly shows that the maximum specific surface area was recorded in PS, followed by PE and PMMA.

### 3.2. Adsorption Behavior between MC-LR, IOM, and MPs

#### 3.2.1. Adsorption Kinetics

**Figure 1:**
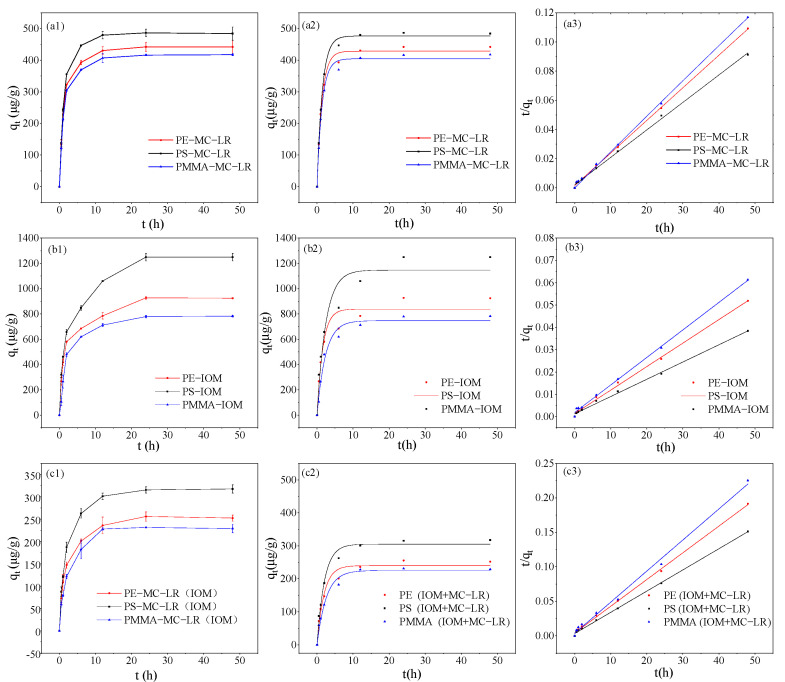
Effect of contact time on adsorption of MC-LR (**a1**), IOM (**a1**–**b3**), and MC-LR in the presence of IOM (**c1**) on PE, PS, and PMMA, respectively, and the fitting graphs of adsorption kinetic models: Pseudo-first-order (**a2**–**c2**) and Pseudo-second-order (**a3**–**c3**).

**Figure 2:**
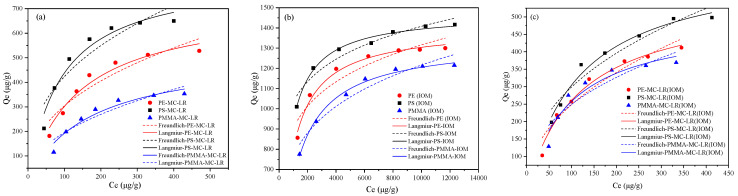
Isothermal adsorption model fitting curves for MC-LR (**a**), IOM (**b**), and MC-LR adsorption in the presence of IOM (**c**) on PE, PS, and PMMA.

**Figure 3:**
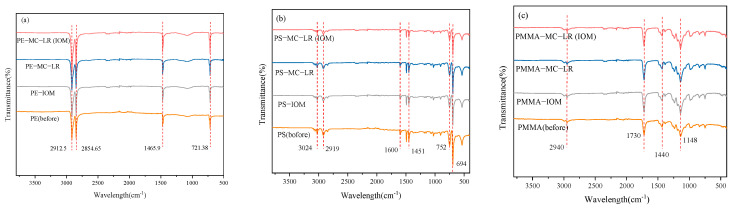
FTIR spectra of (**a**) PE; (**b**) PS, and (**c**) PMMA before and after adsorption of MC-LR and IOM.

The performance of MC-LR, IOM, and MC-LR on adsorbents in the presence of IOM as a function of contact time is illustrated in Figure 1, Figure 2 and Figure 3. The adsorption capacity of PE, PS, and PMMA for MC-LR uniformly increased rapidly in the first 6 h of the adsorption (Figure 1), and the absorbed MC-LR accounted for 88.89%, 92.27%, and 88.52% of the total MC-LR that PE, PS, and PMMA can absorb, respectively. However, as the adsorption time increased, the adsorption efficiency gradually slowed down. After 12 h incubation, the adsorption amount displayed an insignificant increase (*p* > 0.05). Based on the results presented above, a contact time of 24 h was selected for subsequent experiments. The Pseudo-first-order and Pseudo-second-order kinetic models were used to fit the adsorption data (Table S3). The data of the fitting parameters indicated that the MPs were well matched by both the Pseudo-first-order curve (R^2^: 0.992–0.997) and Pseudo-second-order model (R^2^: 0.998–0.999), and the difference between the R^2^ values of the two models was small (Table S1).

The adsorption of IOM on the studied MPs seemed slower than MC-LR. As a function of contact time, the adsorption of IOM on PS occurred quickly within the first 12 h, and the adsorption equilibrium was sufficiently reached after 24 h. The PS seemed to exhibit the most efficient adsorption capability, followed by PE and PMMA. According to the R^2^ values (Table S2), the Pseudo-second-order model fit better than the Pseudo-first-order model.

In the presence of IOM, the adsorption profile of MC-LR on PE, PS, and PMMA was not obviously changed in comparison to that without IOM. However, in addition to the slower adsorption rates (as the equilibrium state was reached within 24 h, but at 12 h for MC-LR adsorption by the MPs with and without the presence of IOM, respectively), the maximum amount of MC-LR absorbed by the three kinds of MPs was substantially reduced at the initial concentration of 400 µg/L MC-LR. These results were in disagreement with previous studies, which found that montmorillonite (clay minerals) adsorbed a moderate amount of fulvic acid, thereby enhancing MC-LR adsorption to Na-montmorillonite [27]. It should be noted that the adsorption of organic matter to an interface could decrease the sorption of organic pollutants due to competition for adsorption sites, or enhance it through increasing the surface hydrophobicity of the particles [28]. Considering the IOM and MC-LR shared the same adsorption process, it was likely that competition for active sites occurred when the MC-LR and IOM coexisted in the solution.

#### 3.2.2. Adsorption Isotherms

The adsorption isotherms results showed that the adsorption amount of MC-LR (Figure 2a) and IOM (Figure 2b) increased with the initial concentrations. The adsorption rate of MC-LR was fast when the toxin concentration was in the range of 50 to 300 µg/ L. When the concentration of MC-LR increased, the adsorption seemed slow down. Figure 2c also revealed that the isotherm curve of MC-LR by MPs in the presence of IOM tended to stabilize after an initial marked increase. A general comparison of the results indicates that in the presence of IOM, the adsorption capacity of MPs for MC-LR is substantially reduced.

The adsorption data were fitted by the Langmuir and Freundlich models and the parameters are presented in Table 2. The results show that MC-LR can be better fitted by the Langmuir model based on the R^2^ values (0.933–0.951), which suggests that the MC-LR sorption onto PE, PS, and PMMA involved monolayer adsorption processes occurring at homogeneous adsorption sites. Similarly, the Langmuir model is more suitable for description of the IOM adsorption by PE, PS, and PMMA, as the correlation coefficient R^2^ was in the range of 0.982 to 0.992. The presence of IOM did not influence the isotherm model of MPs towards MC-LR (better fitting for the Langmuir model, as the R^2^ values were in the range of 0.923 to 0.979, which was higher that the R^2^ of the Freundlich models). However, the adsorption capacity of MC-LR by PE, PS, and PMMA in the presence of IOM decreased by 22.3%, 22.7%, and 5.4%, respectively, in comparison to that without. In addition, of the studied MPs, PS exhibited the greatest adsorption capability toward both MC-LR and IOM, with the adsorption capability reaching 843 and 1470 μg/g, respectively. Recently, it was demonstrated that the hydrophilic MC-LR [29] had lower adsorption onto PE and PS compared to the more hydrophobic Microcystins variant MC-LF [16]. However, the current study revealed that the adsorption capability of MC-LR to hydrophilic PMMA accounted for 61.8% of that on PS. This may be due to the minimum specific surface area of PMMA (0.118 m^2^/g) between the studied MPs (5.136 and 1.281 m^2^/g for PE and PS, respectively). Additionally, the degree of crystallinity of the MPs can also affect the adsorption of MPs. Generally, the degree of crystallinity displayed a negative relationship with the permeability of the polymer. The higher crystallinity of PMMA (Table 1) than PS suggested that PMMA should have a lower adsorption capability towards organic pollutants. It should be noted that the crystallinity of hydrophobic PE (35%) ranked the maximum between the studied MPs, while its adsorption of MC-LR and IOM was greater than for PMMA. This may be linked to the fact that the PE displayed an approximately 10.67-fold greater specific surface area than that of PMMA.

### 3.3. Interaction Mechanism

As can be seen from Figure 3a, the obvious peaks at 2912.5 and 2854.65 cm^−1^ were assigned to CH stretching in –CH_2_– groups [30,31]. The peak at 1465.9 cm^−1^ represented the bending vibrations of the CH_2_ and CH_3_ aliphatic groups [32], while the peak at 721.38 cm^−1^ was ascribed to the –(CH_2_)n– in-plane oscillating vibration [33]. A comparison of the bands between the pristine PE and that of adsorbed MC-LR or IOM showed that the band intensity at 2912.5 and 1465.9 cm^−1^ were stronger when PE adsorbed MC-LR or IOM than the pristine one, indicating that MC-LR and IOM was adsorbed onto PE. However, no new chemical bonds were formed during the adsorption; this suggested that adsorption did not produce a new substance. The peaks of PS can be described at 3024 cm^−1^, which represented the bond-stretching vibrations between the carbon of the aromatic ring and hydrogen, and the peak at 1600 and 1451 cm^−1^ represented the deformation vibration of the C=C, and at 2919 cm^−1^ was attributed to the asymmetric stretching vibration of aliphatic C–H, respectively [34]. The peak at 698 and 752 cm^−1^ was ascribed to the bending C–H of mono-substituted benzene derivatives [35]. In the PMMA spectra (Figure 3c), a relatively broad peak at wavelengths of 2830–2940 cm^−1^, a sharp and intense peak at 1730 cm^−1^, a moderate peak at 1440 cm^−1^, and a peak at 1148 cm^−1^ represented –CH_2_, C=O, O–CH_3_, and C–O bonds, respectively [36]. It can be observed from Figure 3a–c that the peaks were basically similar, with only some adsorption peaks differing slightly, and no new functional groups were detected after the adsorption of MC-LR or IOM by the three MPs, which suggested that physical interaction was the dominant process [37].

**Figure 4:**
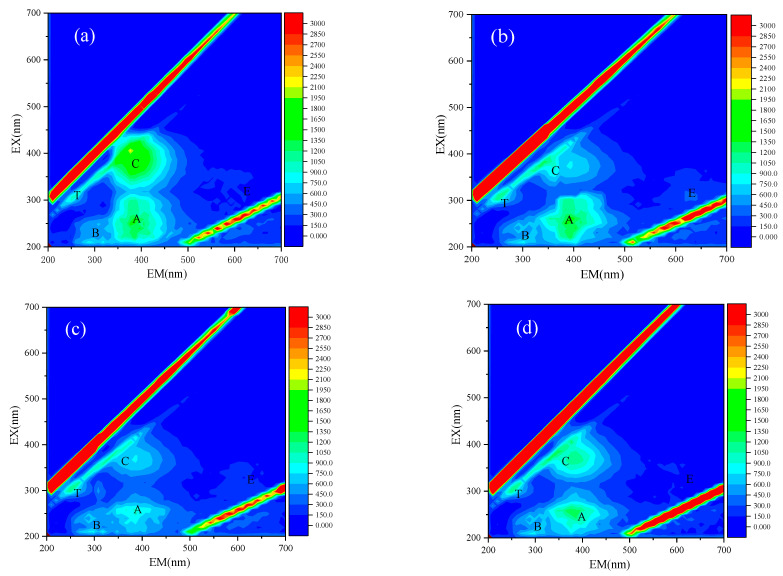
Fluorescence 3D-EEM for IOM after MC-LR adsorption in the presence of IOM in control (**a**), PE (**b**), PS (**c**), and PMMA (**d**).

In the IOM (Figure 4a), peak T at the Ex/Em of 270–275/278–356 nm was identified as soluble microbial metabolites. Peak B (Ex/Em of 210–230/310–344 nm) was considered to be protein-like substances. Peak A (Ex/Em 250–300/400–500 nm) represented fulvic acid-like substances, and peak C (Ex/Em 350–400/400–500 nm) represents humic-like substances [38]. With respect to the adsorption of IOM by PE, the peak in fluorescence from humic-like substances decreased dramatically (Figure 4b). The most obvious alternation in the fluorescence peaks was recorded when PS was used as adsorbent, in which both the fulvic acid-like substances and the humic-like substances were substantially reduced (Figure 4c). Despite PMMA showing affinity to the substance at both peak A and C (Figure 4d), these peaks did not decrease significantly in comparison to PE and PS. The peaks detected in this study disclosed that a considerable amount of IOM was adsorbed by PS, while the humic-like substances might be more easily adsorbed by PE.

**Figure 5:**
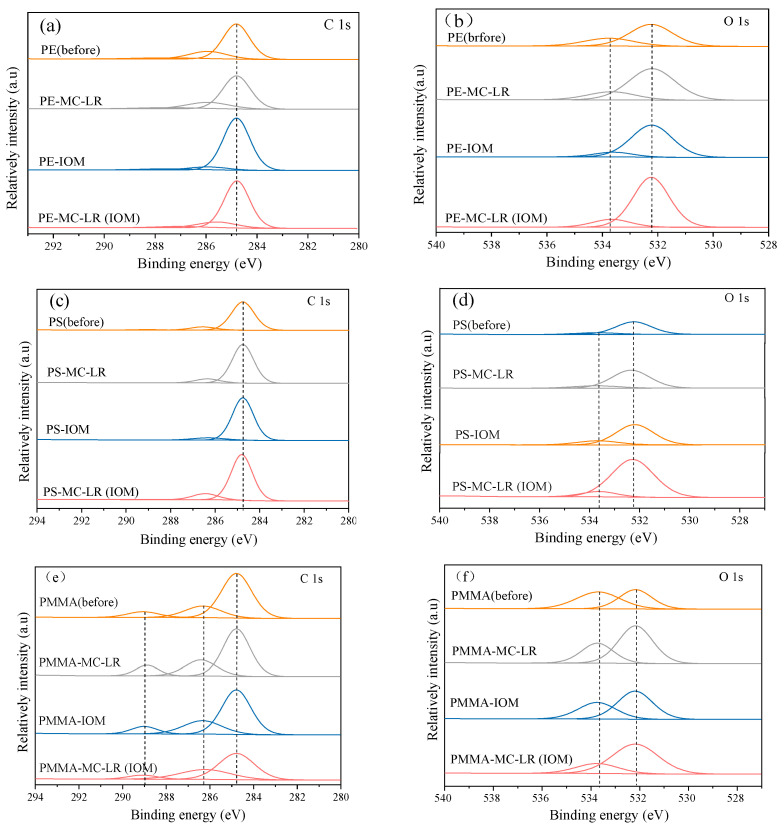
XPS spectra of C, O elements on PE, PS, and PMMA before and after adsorption. C1s (**a**) and O1s (**b**) on PE, C1s (**c**) and O1s (**d**) on PS, C1s (**e**) and O1s (**f**) on PMMA.

The full survey XPS spectra revealed that the studied MPs contained the elements C, N, and O (Figure S4), and the peak of N1s displayed insignificant changes. C1s peaks can be fitted into two components at 284.7 and 286.0 eV, respectively, representing C–C/C–H and C–Cl [39]. According to the XPS spectrum (Figure 5), the peaks of C1s at 286 eV in PE that absorbed IOM were relatively lower, but at 284.8 eV, were stronger than the pristine PE and absorbed MC-LR. The O1s of MPs can be divided into two parts at 532.8 and 533.8 eV, corresponding to –OH and C–O–C/–O–C=O groups, respectively [40], which was substantially enhanced in PE that adsorbed MC-LR in the presence of IOM. A similar pattern was also observed for PS and PMMA. The difference in oxygen content in the three MPs may be caused by the different additives contained in the MPs as well as the adsorbed substance. Table S4 shows the oxygen and carbon content obtained from the wide-scan XPS analysis. The results show that the carbon-to-oxygen (C/O) ratio for PE increased after adsorption, while a dramatic reduction was recorded for PS and PMMA. It should be noted that the oxygen measured in the pristine PE and PS may be due to polymer damage during the blending process, or additives [41]. However, the oxygen content exhibited a dramatic increase after adsorption, indicating the role of oxygen-containing groups in the adsorption process, which contributed to the adsorption [42]. Nevertheless, PMMA had the maximum oxygen content, while displaying the minimum adsorption capability in the studied MPs (Table 2); this may be due to the minimum specific surface area (Table 1).

Table 3 presents the available adsorption mechanism that PE and PS involved (little information was available for PMMA) in the adsorption of organic pollutants. It can be seen from the table that the physical interactions, including partitioning, van der Waals force, electrostatic interactions, and intermolecular hydrogen bonding, were the dominant mechanisms. It can also be concluded from Table 3 that PS exhibited greater adsorption capability towards the same organic chemicals than PE, which was in line with the current study concerning the adsorption of MC-LR and IOM.

**Figure 6:**
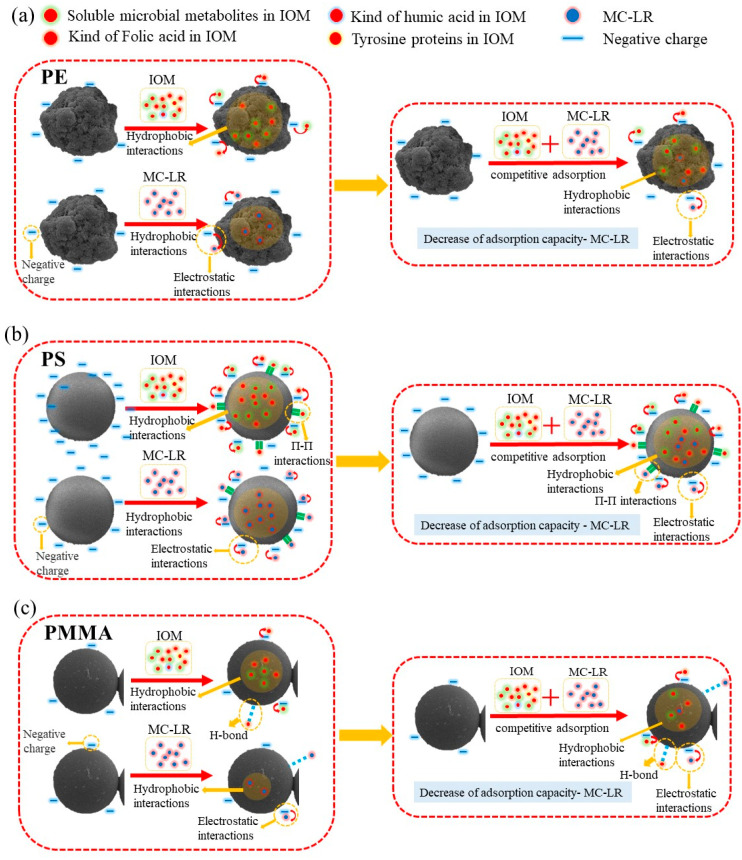
Conceptual schematic of PE (**a**), PS (**b**), and PMMA (**c**) adsorption of MC-LR and IOM, as well as the adsorption process of MC-LR in the presence of IOM.

In the current study, the FTIR results showed that no new chemical bonds were formed during the adsorption of MC-LR by the three MPs, suggesting that physical adsorption may play a crucial role in the adsorption process. According to the zeta potential of the three kinds of MPs (Figure S1), PE, PS, and PMMA had negative inherent charges (<0 mV at pH in the range between 4.5 and 9), indicating that electrostatic repulsion may inhibit the adsorption of MC-LR, since the toxin was negatively charged at most pH values (3 < pH < 12) [37]. Apart from that, the van der Waals force and pore filling effect may be involved in the physical adsorption of MC-LR by the three kinds of MPs. In this study, the crystallinity of PE reached 35%, which may hinder the mass transfer of MC-LR due to its low permeability. The IOM displayed higher affinity towards PE, as revealed by the increased relative intensity in C1s (284 eV) (Figure 5a) and the substantial reduction of humic-like substances during the adsorption of IOM (Figure 5b). Therefore, it was highly possible that the co-existence of IOM competed the active sites between MC-LR (Figure 6a); thus, the MC-LR adsorption capability was reduced upon the presence of IOM (Table 2). In terms of PS, the benzene structure and the specific surface area may contribute to the maximum adsorption capability towards both MC-LR and IOM. In addition, it is possible that Π-Π bonds strengthened the interactions between PS and the chemicals; this agreed well with a recent study that investigated the adsorption of hydrophilic organic chemicals by PS [37]. This structure could also increase the polarity of PS, which prompted its interaction with IOM, as revealed by the substantial reduction of fulvic acid-like substances and humic-like substances that originated from IOM (Figure 4c). Consequently, the increased competition for binding sites between MC-LR and IOM reduced its adsorption capability once the IOM was prevalent in the surrounding environment (Figure 6b). PMMA displayed hydrophobicity and was anticipated to adsorb more hydrophilic MC-LR than PE and PS, while its C=O facilitated its conjunction with the hydroxyl and carboxyl in the IOM through hydrogen bonding; thus, the adsorption of MC-LR can be depressed (Figure 6c). This was supported by the XPS and 3D-EEM analysis. The multiple processes of the adsorption of MC-LR and IOM by PE, PS, and PMMA, as well as the influence of IOM on MC-LR adsorption, are illustrated in Figure 6.

### 3.4. Potential Environmental Impact

MPs are receiving increasing attention since they can act as a vector for a variety of pollutants, which can enhance risk through the transfer of pollutants along the food chain. In this study, we evaluated the adsorption of pollutants of natural origin (MC-LR), which may be accompanied by other chemicals that likely influence the adsorption, by three prevalent MPs of uniform size but differences in hydrophobicity. The results documented that PE, PS, and PMMA adsorbed MC-LR, with the maximum adsorption capability recorded in PS. This observation manifested the potential risk of MPs as vector for the cyanotoxin. However, since MC-LR is released into the surrounding water as a consequence of cell rupture or the self-dependence of toxin-producing Microcystis, the toxin is always detected in the natural water associated with the substantial increase of dissolved organic matter. Apart from the affinity of MC-LR towards the three kinds of studied MPs, IOM was also adsorbed onto the MPs, which increased competition for available binding sites between MC-LR, during which multiple physical processes were involved. This suggested that the batch experiment used to assess the role of MPs as pollutants of natural origin may be magnified. In addition, in our recent study, we found that PS absorbed MC-LR and favored the colonization of microcystin-degrading bacteria; thus, the biodegradation of the toxin was identified [25]. As a result, our previous and current study indicates that the potential risk and environmental impact of MPs should be evaluated under conditions that could more closely resemble the real scenario, which requires further multidisciplinary investigations.

## 4. Conclusions

In this study, we evaluated the adsorption of three kinds of microplastics towards toxic chemicals of natural origin (MC-LR), and investigated the influence of intracellular organic matter (IOM), which was released into the surrounding water, accompanied by MC-LR, by toxin-producing Microcystis, on the adsorption of MC-LR by MPs. We then identified the potential mechanism involved in the adsorption. We found that PE, PS, and PMMA, with the uniform size of 50 µm in diameter, displayed high affinity toward MC-LR, with adsorption capabilities of 722, 843, and 521 μg/g, respectively. This was caused by physical adsorption processes including van der Waals force, electrostatic interaction, and pore filling. In addition, the maximum adsorption of MC-LR was also attributed to the formation of Π-Π bonds that strengthened the interactions between PS and MC-LR, as well as the maximum specific surface area displayed by PS. Apart from that, the PE, PS, and PMMA could also adsorb IOM, with the adsorption capability of 1400 μg/g for PE, 1470 μg/g for PS, and 1300 μg/g for PMMA. This indicated that a higher affinity was shown towards IOM by the PE, PS, and PMMA in comparison to MC-LR. However, the increased competition for binding sites between MC-LR and IOM reduced the adsorption of MC-LR by PE and PS. Despite the PMMA displaying hydrophobicity, its C=O facilitated its conjunction with the hydroxyl and carboxyl in the IOM through hydrogen bonding; thus, the adsorption of MC-LR was depressed by 5.4% when the MC-LR and IOM co-existed in the solution. Given the potential role of microplastics as vectors of contaminants in the environment, the results of this study provide new insights to assess its ability to carry chemicals of natural origin.

## Figures and Tables

**Table 1:** Structure and basic physical and chemical characters of PE, PS, and PMMA.

MPs	Molecular Formula	Structure	d_(0.5)_ (μm)	Specific Surface Area (m^2^/g)	Contact Angles (°)	Crystallinity (%)
PE	(C_2_H_4_)_n_	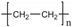	43.06	1.281	108.8 ± 3.8	35
PS	(C_8_H_8_)_n_	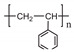	49.96	5.136	106.3 ± 2.3	3.7
PMMA	(C_5_H_8_O_2_)_n_	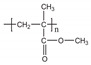	55.47	0.118	85.1 ± 1.9	6.1

**Table 2:** Adsorption isotherm parameters of MC-LR and IOM on the studied MPs.

MPs	Adsorbate	Freundlich				Langmuir	
		*k_f_*	1/*n*	*R* ^2^	*q_m_* (μg/g)	*k_a_*	*R* ^2^
PE	MC-LR	45.1	2.40	0.857	722	0.00719	0.951
	IOM	293	6.13	0.868	1400	0.00132	0.982
	MC-LR (+IOM)	30.5	2.19	0.920	558	0.00875	0.979
PS	MC-LR	75.1	2.66	0.837	843	0.0108	0.946
	IOM	409	7.46	0.930	1470	0.00176	0.988
	MC-LR (+IOM)	46.2	2.47	0.924	655	0.00867	0.979
PMMA	MC-LR	23.2	2.16	0.853	521	0.00584	0.933
	IOM	191	4.98	0.935	1330	0.000940	0.992
	MC-LR (+IOM)	41.3	2.56	0.808	493	0.0110	0.923

Note: +IOM indicates the MC-LR adsorption in the presence of IOM.

**Table 3:** Comparison of the adsorption capability and adsorption mechanism towards organic pollutants of the studied MPs.

MP Type	MP Size	Organic Pollutants	Adsorption Amount (μg/g)	Adsorption Mechanism	References
PS PE	200 ± 10 μm	17β-estradiol	92.4 86.3	hydrogen bonds and π–π interaction	[43]
PE	0.125–0.425 mm	Tri-n-butyl phosphate	1.426	pore–filling, monolayer coverage	[44]
		Tris(2-chloroethyl) phosphate)	0.532		
PS	50.4 ± 11.9 μm	Atorvastatin	1610	hydrophobic and π–π interaction	[45]
		Amlodipine	460		
PE	<5 mm	Carbendazim	4.444	hydrophobic interactions	[46]
		Dipterex	2.873		
		Diflubenzuron	74.129		
		Malathion	25.907		
		Difenoconazole	273.224		
PE	0.71–0.85 mm	Imidacloprid	2.630	surface adsorption	[47]
		Buprofezin	1.892		
		Difenoconazole	2.365		
PS PE	0.5–1 mm 0.1–0.2 mm	Cephalosporin C	709 717	hydrophobic interaction, van der Waals force, and electrostatic interactions	[48]
PE	100 μm	Ciprofloxacin	5852	hydrophobic interaction and electrostatic interactions	[49]
PE PS	<200-mesh	Tylosin	1666.67 3333.33	electrostatic interactions, hydrophobic interactions, and surface complexation	[50]
PS	0.45–1 mm	Oxytetracycline	1520 ± 120	hydrophobic interaction or hydrogen bonding	[51]
PE	<0.15 mm	3,6-dibromocarbazole	15.3 ± 3.57	chemical sorption	[52]
		3,6-dichlorocarbazole	24.8 ± 3.95		
		3,6-diiodocarbazole	118 ± 42.3		
		2,7-dibromocarbazole	16.6 ± 1.15		
		3-bromocarbazole	17.1 ± 1.85		
PE PS	100–150 μm	Sulfamethoxazole	660 712	hydrogen bond	[53]
PS	100 μm	Triadimenol	34.36	hydrophobic and electrostatic interactions	[54]
		Hexaconazole	185.18		
PE	150 μm	Carbofuran	10,729.6	van der Waals force	[55]
		Carbendazim	5458.5		
PE PS	150 μm, <280 μm	Tetracycline	109 ± 3.62, 167 ± 7.74	hydrophobic interactions and other interactions (e.g., electrostatic interactions)	[56]
PE PS	100–150 μm	Pyrene	333 127	monolayer coverage	[57]
PE PS	25 μm	Norfloxacin	444 758	π–π conjugation, hydrogen bonds, ion exchange, and electrostatic interactions	[58]
PS * PE	550 μm	Benzophenone-3	53.193 *, 26.382	liquid film diffusion and intraparticle diffusion	[59]
	250 μm		62.544 *, 38.807		
	75 μm		78.609 *, 41.142		
	5 μm		89.291 *, NA		
	0.5 μm		97.559 *, NA		
PE	0.15–0.425 mm	Chlortetracycline hydrochloride	355.5	intermolecular van der Waals force	[60]
		Oxytetracycline hydrochloride	352.6		
		Tetracycline hydrochloride	253.9		
PS	~75 μm	Ciprofloxacin	10,200	partition, hydrogen bonding, and electrostatic interaction	[37]
PE	28 μm	Ofloxacin	40.8	partitioning and van der Waals force	[61]
	48 μm		15.2		
	75 μm		6.9		
	250 μm		1.8		
	590 μm		1.4		
	28 μm	Levofloxacin	39.5		
	48 μm		13.6		
	75 μm		5.6		
	250 μm		1.4		
	590 μm		1.1		
PE	50 μm	MC-LR	722	van der Waals force, electrostatic interaction and pore-filling	This study
PS	50 μm	MC-LR	843	van der Waals force, electrostatic interaction, and pore–filling, π–π bond	
PMMA	50 μm	MC-LR	521	van der Waals force, electrostatic interaction and pore-filling, hydrogen bond	

Note: NA means the the data didn’t acquired; * indicates the obtained adsorption capability in corresponding to the MPs mentioned.

## Data Availability

Not applicable.

## Supplementary Materials

The supplementary materials include the characterization of the three kinds of MPs in this study, including particle size distribution, zeta potential, XRD patterns, and microscopic images of contact angles and SEM micrographs; the XPS spectra of a survey scan of the MPs before and after adsorption; and the adsorption kinetics fitting parameters and the carbon and oxygen content in the MPs before and after adsorption, obtained through wide-scan XPS analyses. The following supporting information can be downloaded at: https://www.mdpi.com/article/10.3390/toxics10060339/s1, Figure S1: (**a**) Particle size distribution; (**b**) zeta potential of the studied microplastics; Figure S2: XRD patterns of (**a**) PE, (**b**) PS, and (**c**) PMMA; Figure S3: Microscopic images of contact angles (**a**) and SEM micrographs (**b**) of PE, PS, and PMMA 19 (magnification of 500×, 2000×); Figure S4: XPS spectra of survey scan of (**a**) PE, (**b**) PS, and (**c**) PMMA before and after adsorption; Table S1: Adsorption kinetics fitting parameters of MCs on microplastics; Table S2: Adsorption kinetics fitting parameters of IOM on microplastics; Table S3: Adsorption kinetics fitting parameters of MCs on microplastics in the presence of IOM; Table S4: Carbon and oxygen content in the MPs before and after adsorption, obtained through wide-scan XPS analyses.
