# Supplementary material for: The Inhibition of Microcystin Adsorption by Microplastics in the Presence of Algal Organic Matters
Source: Toxics. 2022 Jun 20;10(6):339. doi: 10.3390/toxics10060339 (PMC9230722; doi:10.3390/toxics10060339)
Supplement: Supplementary file 1 [file toxics-10-00339-s001.zip › toxics-1757712-supplementary.pdf]

__Supporting information__

The inhibition of microcystin adsorption by microplastics in the presence of algal organic matters

Bingran Tang [1], Ying Tang [2], Xin Zhou [1], Mengzi Liu [1], Hong Li [1,*] and Jun Qi [3,*]

1 Key Laboratory of Eco-Environment of Three Gorges Region, Ministry of Education, Chongqing University, Chongqing 400044, China; tangbingrancqu@163.com (B.T.); zhouxincqu@163.com (X.Z.); liumengzicqu@sina.com (M.L.)

2 Chongqing Key Laboratory of Soil Multi-Scale Interfacial Process, Department of Soil Science, College of Re-sources and Environment, Southwest University, Chongqing 400715, China; yingtang@swu.edu.cn

3 Department of hepatobiliary pancreatic tumor center, Chongqing University Cancer Hospital, Chongqing 400045, China

* Correspondence: hongli@cqu.edu.cn (H.L.); yunshen315@13.com (J.Q.)

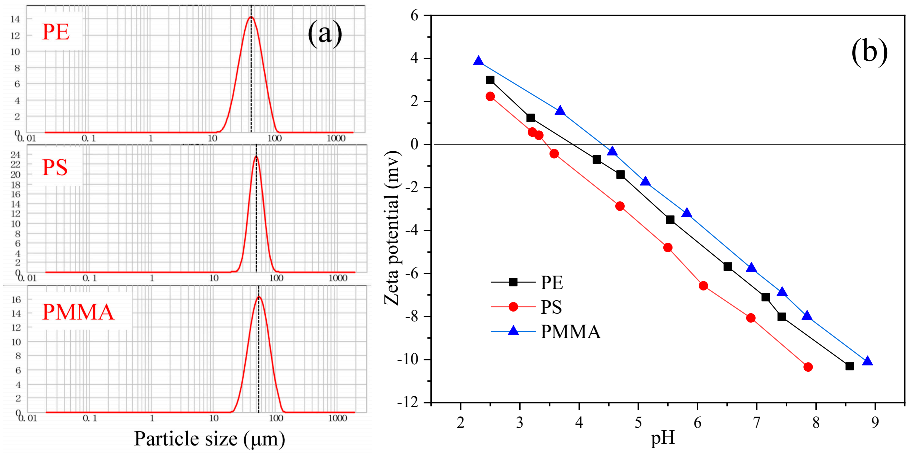

Figure S1 (a) Particle size distribution; (b) Zeta potential of the studied microplastic

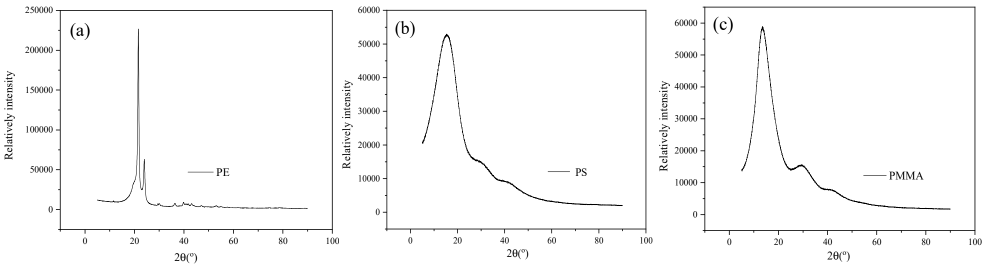

Figure S2 XRD patterns of (a) PE; (b) PS and (c) PMMA.

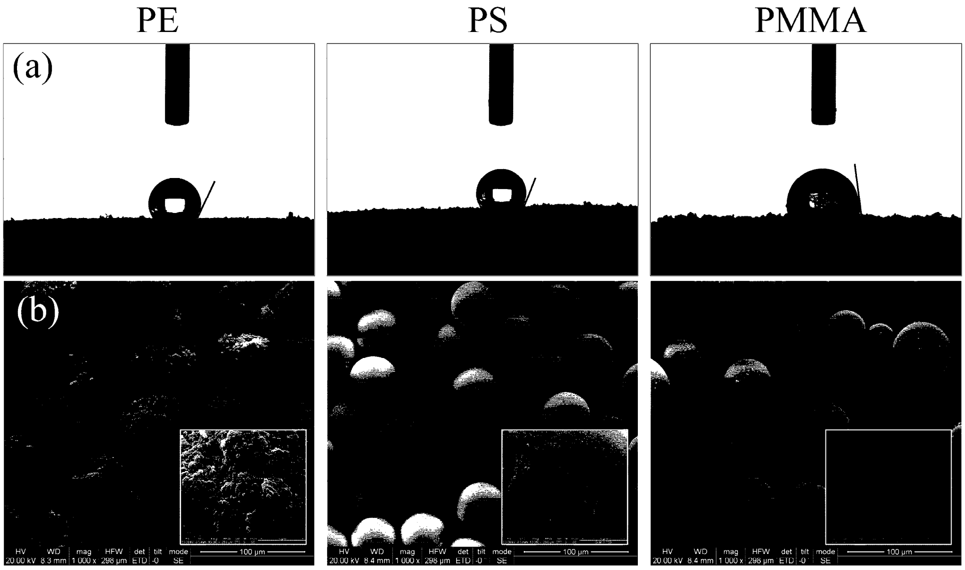

Figure S3 Microscopic images of contact angles(a) and SEM micrographs(b) of PE, PS and PMMA (magnification of 500×、2000×).

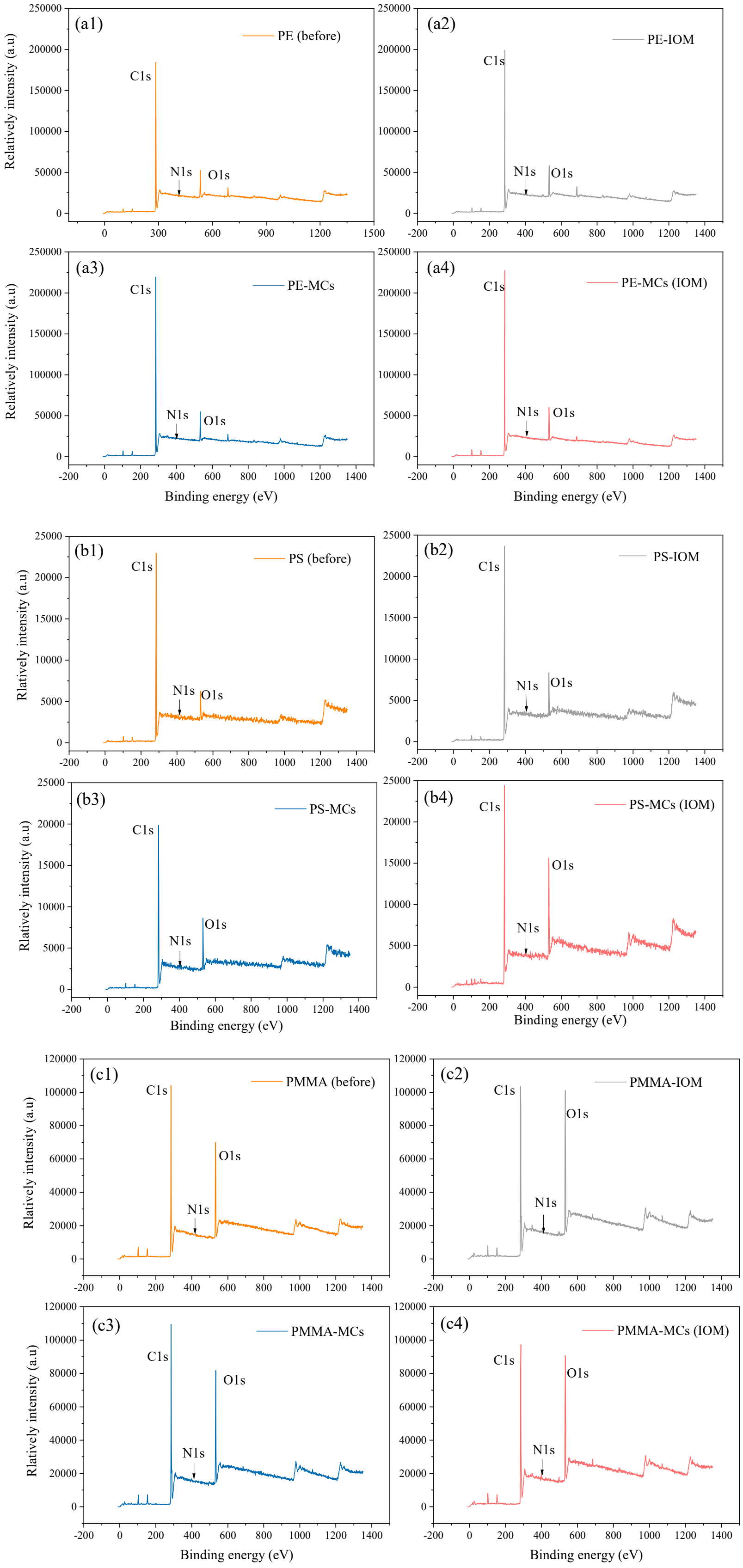

Figure S4 XPS spectra of survey scan of (a) PE; (b) PS and (c) PMMA before and after adsorption.

Table S1 Adsorption kinetics fitting parameters of MCs on microplastics

MP Type	Pseudo first order			Pseudo second order		
	q_e (μg/g)	K_1(h^{-1})	R^2	q_e(μg/g)	K_2(g/μg/h)	R^2
PE	429	0.726	0.993	448	0.00319	0.999
PS	477	0.689	0.997	503	0.00255	0.998
PMMA	404	0.711	0.992	429	0.00290	0.998

Table S2 Adsorption kinetics fitting parameters of IOM on microplastics

MP Type	Pseudo first order			Pseudo second order		
	q_e (μg/g)	K_1(h^{-1})	R^2	q_e(μg/g)	K_2(g/μg/h)	R^2
PE	839	0.625	0.948	952	0.000793	0.998
PS	1150	0.409	0.941	1290	0.000456	0.997
PMMA	747	0.424	0.982	813	0.000800	0.998

Table S3 Adsorption kinetics fitting parameters of MCs on microplastics at the presence of IOM

MP Type	Pseudo first order			Pseudo second order		
	q_e (μg/g)	K_1(h^{-1})	R^2	q_e(μg/g)	K_2(g/μg/h)	R^2
PE	240	0.520	0.972	258	0.00351	0.999
PS	305	0.488	0.986	326	0.00272	0.999
PMMA	225	0.391	0.983	222	0.00520	0.996

Table S4 Carbon and oxygen content in the MPs before and after adsorption obtained through wide-scan XPS analyses.

MPs	Polymer Type	C (%)	O (%)	C/O
PE	PE	91.1	8.48	10.74
	PE-IOM	89.69	9.5	9.44
	PE-MC-LR	91.65	7.79	11.77
	PE- MC-LR (IOM)	90.82	8.6	10.56
PS	PS	91.05	7.92	11.50
	PS-IOM	89.51	10.49	8.53
	PS- MC-LR	86.88	11.16	7.78
	PS- MC-LR (IOM)	78.5	20.39	3.85
PMMA	PMMA (Before)	80.01	19.54	4.09
	PMMA-IOM	74.72	24.63	3.03
	PMMA- MC-LR	77.73	21.38	3.64
	PMMA- MC-LR (IOM)	76.13	22.45	3.39
